# Repurposing of approved cardiovascular drugs

**DOI:** 10.1186/s12967-016-1031-5

**Published:** 2016-09-20

**Authors:** Junichi Ishida, Masaaki Konishi, Nicole Ebner, Jochen Springer

**Affiliations:** Innovative Clinical Trials, Department of Cardiology and Pneumology, University Medical Centre Göttingen, Robert-Koch-Str. 40, 37075 Göttingen, Germany

**Keywords:** Drug repurposing, Drug repositioning, Cardiovascular drugs, Second label indication, Pleiotropic properties

## Abstract

Research and development of new drugs requires both long time and high costs, whereas safety and tolerability profiles make the success rate of approval very low. Drug repurposing, applying known drugs and compounds to new indications, has been noted recently as a cost-effective and time-unconsuming way in developing new drugs, because they have already been proven safe in humans. In this review, we discuss drug repurposing of approved cardiovascular drugs, such as aspirin, beta-blockers, angiotensin converting enzyme inhibitors, angiotensin II receptor blockers, cardiac glycosides and statins. Regarding anti-tumor activities of these agents, a number of experimental studies have demonstrated promising pleiotropic properties, whereas all clinical trials have not shown expected results. In pathological conditions other than cancer, repurposing of cardiovascular drugs is also expanding. Numerous experimental studies have reported possibilities of drug repurposing in this field and some of them have been tried for new indications (‘bench to bedside’), while unexpected results of clinical studies have given hints for drug repurposing and some unknown mechanisms of action have been demonstrated by experimental studies (‘bedside to bench’). The future perspective of experimental and clinical studies using cardiovascular drugs are also discussed.

## Background

Drug repurposing is a way to identify a new indication for existing drugs and compounds and is also called as drug repositioning, drug rescue or drug re-profiling. Drug repurposing generates lower costs and shorter time until approval than developing a drug de novo, because all phases of clinical trials have already performed for approved drugs and the information regarding side effects, pharmacokinetics and interaction with other drugs has been collected. Drug repurposing could also be useful for the treatment of rare or orphan diseases without any proven treatments [1].

In this review, we discuss drug repurposing of approved cardiovascular drugs, such as aspirin, beta-blockers, angiotensin converting enzyme (ACE) inhibitors, angiotensin II receptor blockers (ARBs), cardiac glycosides and statins, which are commonly prescribed for the treatment and/or prevention of cardiovascular diseases. We focus on pleiotropic properties and action mechanisms of each agent in tumor progression and metastasis. Aspirin not only prevents platelet function but also suppresses tumor cell proliferation directly. Beta-blockers inhibit tumor angiogenesis and invasiveness and induce apoptosis. ACE inhibitors and ARBs suppress tumor growth, angiogenesis and invasiveness. Pro-apoptotic activity of cardiac glycosides and anti-proliferative activity of statins are also described. Subsequently, we summarize the findings of clinical studies investigating the relationship between cardiovascular drugs and cancer. Cancer-related events have not been included in the primary endpoints in most of the large-scale cardiovascular clinical trials, which makes it difficult to evaluate whether cardiovascular drugs really exhibit anti-tumor effects identified in experimental research. Nevertheless, aspirin and beta-blockers have advanced to randomized clinical trials (RCTs) to confirm their anti-tumor effects, while findings have been inconsistent with regard to cancer and ACE inhibitors, ARBs, cardiac glycosides or statins.

Repurposing of cardiovascular drugs is also expanding in pathological conditions other than cancer.

Numerous experimental studies have reported possibilities of drug repurposing in the cardiovascular field and some of them have been tried for new indications (‘bench to bedside’), whereas unexpected results of clinical studies have given hints for drug repurposing and the unknown mechanisms of action have been demonstrated by experimental studies (‘bedside to bench’). The excellent examples of drug repurposing are propranolol for infantile hemangioma, beta-blockers for migraine, preoperative statins for perioperative risk reduction and minoxidil for androgenic alopecia, which are prescribed in daily clinical practice. Beta-blockers might be effective for the treatment of cirrhosis and osteoporosis. RCTs are examining whether losartan and statins are effective for Marfan’s syndrome and contrast-induced nephropathy, respectively. In addition to the available knowledge, the future perspective of experimental and clinical studies using cardiovascular drugs are also discussed.

## Repurposing of cardiovascular drugs in cancer

### Aspirin and cancer

**Fig. 1:**
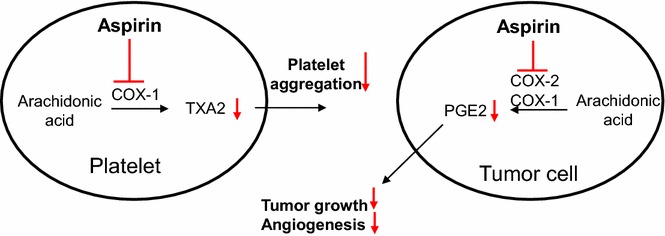
Putative mechanisms of action of low-dose aspirin in platelets and tumor cells in suppressing tumor growth. Low-dose aspirin exerts an inhibitory effect on platelet aggregation by suppressing production of TXA2 through inhibition of COX-1 in platelets. Thus, low-dose aspirin prevents platelets from binding to tumor cells, resulting in suppression of distant metastasis. On the other hand, PGE2, which is upregulated in colon cancer cells, is suppressed by low-dose aspirin, leading to inhibition of tumor growth and angiogenesis. *COX-1* cyclooxygenase-1, *COX-2* cyclooxygenase-2, *PGE2* prostaglandin E2, *TXA2* thromboxane A2

Aspirin is commonly used for the treatment and prevention of atherosclerotic diseases, and pharmacological targets of aspirin are two isoforms of cyclooxygenase (COX) enzyme, COX-1 and COX-2 [2]. COX-1 is a constitutive enzyme expressed in most mammalian tissues and produces thromboxane A2 (TXA2) in platelets, which promotes platelets aggregation and adherence of platelets to tumor cells and thus prevents immune cells from recognizing and eliminating them, resulting in increased distant metastasis. On the other hand, COX-2 is a rapidly inducible enzyme during inflammation and dominantly produces prostaglandin E2 (PGE2) in tumor cells compared with COX-1, and PGE2 is thought to play an important role in accelerating cell proliferation and tumor growth. Aspirin administered at low doses (50–100 mg daily) and high doses (>325 mg daily) selectively blocks COX-1 and COX-2 in an irreversible manner, respectively. The putative action mechanisms of aspirin in tumor progression and metastasis are displayed in Fig. 1. Since the anti-tumor effect of aspirin was first reported in tumor-bearing mice in 1972 [3, 4], a number of subsequent experimental studies have supported this evidence [5]. As most clinical trials have shown a significant reduction in cancer risk and cancer-associated death in patients taking aspirin at a low dose, but not a high dose [6, 7], one important mechanism of tumor suppression by aspirin is proposed as inhibition of COX-1. With this activity, low-dose aspirin could prevent platelets from binding to tumor cells, resulting in suppression of distant metastasis and improved survival. In addition, PGE2 was upregulated in colon cancer [8] and administration of PGE2 enhanced tumor growth and angiogenesis [9]. As PGE2 was significantly suppressed in human colons when aspirin was administered even at a low dose (81 mg daily) [10], Suppression of PGE2 might be also important in anti-tumor activity of aspirin.

In clinical fields, a case–control study first demonstrated that aspirin use was associated with reduced risk of CRC (colorectal cancer) (risk ratio (RR) 0.53, 95 % confidence interval [CI] 0.40–0.71, p < 0.001) in 1988 [11]. Since then, a number of observational studies have shown that regular aspirin use significantly reduced risk of several cancers including CRC [12], esophageal cancer [13], gastric cancer [13], breast cancer [13] and prostate cancer [14–16]. In addition, Rothwell et al. reported that regular aspirin use reduced not only risk of distant metastasis [Hazard ratio (HR) 0.64, 95 % CI 0.48–0.84, p = 0.001] [17], but also cancer-related death [Odds ratio (OR) 0.79, 95 % CI 0.68–0.92, p = 0.003] [7]. Regarding the dose and the duration of aspirin, a meta-analysis of the five RCTs showed that aspirin at low dose (75–300 mg daily) reduced the 20-year incidence and mortality of CRC (incidence HR 0.75, 95 % CI 0.56–0.97, p = 0.02; mortality HR 0.61, 95 % CI 0.43–0.87, p = 0.005) and that the effects of aspirin increased with the duration of the treatment [6]. The results of recent meta-analysis are summarized in Table 1. Thus, aspirin could be effective for the prevention and/or the treatment of cancers. However, these findings are based on the results of observational studies and RCTs to evaluate the effects of aspirin on cardiovascular events. In addition, bleeding and gastrointestinal complications should be taken into consideration in the use of aspirin. To investigate the efficacy and safety of aspirin, the Aspirin in Reducing Events in the Elderly (ASPREE; NCT01038583) study, a RCT, is ongoing. Currently aspirin should be administered only for patients with cardiovascular diseases, not for the prevention of cancer.

**Table 1:** Anti-tumor effects of aspirin in recent meta-analyses

Author (year) [reference]	Number of studies (number of patients)	Dose of aspirin (mg)	Type of cancer	Main findings
González-Pérez et al. (2003) [13]	4, 5 and 11	Any	Esophageal, gastric and breast cancer	Aspirin reduced the incidence of esophageal cancer (RR 0·51, 95 % CI 0.38–0.69), gastric cancer (RR 0.73, 0.63–0.84) and breast cancer (RR 0.77, 0.69–0.86), derived from four, five and eleven studies respectively
Flossmann et al. (2007) [12]	2 (5061)	300<	CRC	Aspirin reduced the incidence of CRC (HR 0.74, 95 % CI 0.56–0.97, p = 0.02)
Rothwell et al. (2010) [6]	5	75–300	CRC	Low-dose aspirin reduced the 20-year incidence and mortality of CRC (incidence HR 0.75, 95 % CI 0.56–0.97, p = 0.02; mortality HR 0.61, 95 % CI 0.43–0.87, p = 0.005)
Rothwell et al. (2011) [7]	8	75<	Any	Regular aspirin use reduced cancer-related death (OR 0.79, 95 % CI 0.68–0.92, p = 0.003). Therapeutic effects increased with duration of aspirin use
Rothwell et al. (2012) [17]	5	75<	Any	Regular aspirin use reduced the risk of distant metastasis (HR 0.64, 95 % CI 0.48–0.84, p = 0.001)

### Beta-blockers and cancer

Previous experimental studies have demonstrated that chronic stress, depression and social isolation are associated with tumor progression [18–21]. As catecholamines such as norepinephrine and epinephrine are elevated under chronic stress and their effects are mainly mediated through beta-adrenoreceptors, activation of beta-adrenoreceptors by catecholamines is believed to play an important role in tumor progression. Indeed, the presence of beta-adrenoreceptors has been shown in the cell lines of breast cancer [22], pancreatic cancer [23], nasopharyngeal cancer [24] and ovarian cancer [25], and catecholamines significantly increased cell proliferation as well as cell migration in human cancer cell lines [26, 27]. Furthermore, in a mouse model of ovarian cancer, beta-adrenergic stimulation not only increased angiogenesis and tumor invasion through the cyclic adenosine monophosphate (cAMP)-protein kinase A (PKA) pathway [25], but also prevented the cancer cells from apoptosis by activating focal adhesion kinase (FAK) [28]. Additionally, a recent study showed that beta-adrenergic activation increased distant metastasis by 30 times through M2 macrophage infiltration in a mouse model of breast cancer [29].

**Fig. 2:**
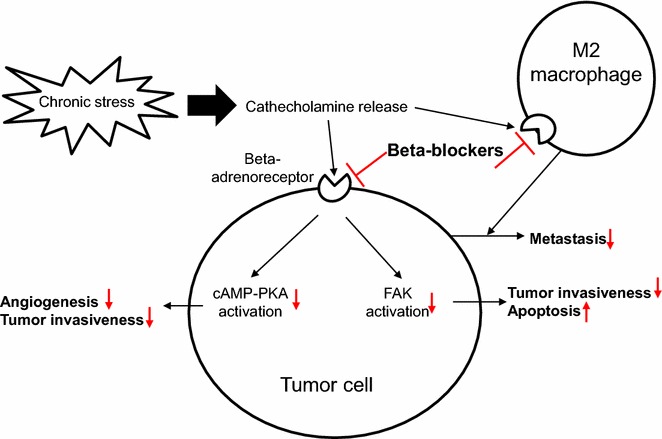
Putative mechanisms of action of beta-blockers in preventing tumor progression. Catecholamines are elevated under chronic stress and bind to beta-adrenoreceptors, resulting in activation of cAMP-PKA pathway and FAK, which accelerates tumor angiogenesis and invasion, and prevents cancer cells from apoptosis respectively. Beta-blockers blocks beta-adrenoreceptors, so that they are believed to suppress tumor growth and invasion. *cAMP* cyclic AMP, *FAK* protein kinase A, *FAK* focal adhesion kinase

On the other hand, beta-blockers mainly block beta-adrenoreceptors, and have been investigated as treatment for malignancies as well as cardiovascular diseases. Generally prescribed beta-blockers are classified into three types depending on selectivity of receptor subtypes, beta-1 selective beta-blockers such as bisoprolol, non-selective beta-blockers (NSBB) such as propranolol and nadolol, and alpha- and beta-blockers such as carvedilol, which block beta-1, all types of, and alpha- and beta-adrenoreceptors respectively. All of these beta-blockers are widely used for the treatment of heart failure, hypertension, ischemic heart disease and arrhythmias in the cardiovascular field. A number of experimental studies have also shown the anti-tumor effects of beta-blockers. In human cancer cell lines, catecholamine-induced proliferation and migration were inhibited by NSBB [26, 27], and enhanced invasiveness [30] and activated FAK by catecholamine were completely blocked by propranolol in a mouse model of ovarian cancer [28]. The action mechanisms of beta-blockers in tumor cells described above are currently proposed, as shown in Fig. 2. Moreover, in a mouse model of breast cancer, propranolol counteracted catecholamine-induced metastasis to distant tissues through M2 macrophage infiltration [29], which indicates the importance of beta-adrenergic signaling in M2 macrophage. Interestingly, a recent publication demonstrated that bisoprolol improved cardiac function and survival in a dose-dependent manner in rats with cancer cachexia, suggesting the favorable effects of beta-blockers in the terminal stage of cancer [31].

In clinical settings, several epidemiological studies have examined the potential effect of beta-blockers on the incidence and the outcome of cancer. The results have been inconsistent [32–37], as shown in Table 2, but some of them demonstrated that the use of beta-blockers was associated with improved overall survival in patients with certain types of cancer such as breast cancer (HR 0.19, 95 % CI 0.06–0.60) [32], ovarian cancer (HR 0.54, 95 % CI 0.31–0.94, p = 0.02) [33] and non-small cell lung carcinoma (HR 0.78, 95 % CI 0.63–0.97, p = 0.02) [34]. In addition, a recent meta-analysis of 12 clinical studies have shown that beta-blocker usage was associated with significantly improved overall survival (HR 0.79, 95 % CI 0.67–0.93, p = 0.004) [38]. Beta-blockers appeared to have a greater effect in patients with early-stage cancer or cancer treated with primary surgery than those with late-stage cancer or cancer treated without primary surgery [38].

**Table 2:** Anti-tumor effects of beta-blockers in recent clinical studies

Authors (year), reference	Number of patients taking beta-blockers	Type of cancer	Main findings
Fryzek et al. (2006) [155]	NA	Breast cancer	The use of beta-blockers was not associated the risk of breast cancer (RR 1.07, 95 % CI 074–1.56)
Assimes et al. (2008) [156]	1788	Any	Beta-blockers significantly reduced the risk of cancer (OR 0.9, 95 % CI 0.85–0.96)
Powe et al. (2010) [157]	43	Breast cancer	Patients taking beta-blockers had a 57 % reduced risk of metastasis (Hazard ratio 0.43, 95 % CI 0.20–0.93)
Barron et al. (2011) [32]	70	Breast cancer	Propranolol reduced cancer-related mortality (HR 0.19, 95 % CI 0.06–0.60)
Ganz et al. (2011) [36]	204	Breast cancer	Beta-blocker usage was not associated with improved overall survival (HR 1.04, 95 % CI 0.72–1.51)
Lemeshow et al. (2011) [37]	275	Melanoma	Beta-blockers reduced all-cause mortality (HR 0.81, 95 % CI 0.67–0.97)
Diaz et al. (2012) [33]	23	Ovarian cancer	Beta-blockers improved overall survival (HR 0.54, 95 % CI 0.31–0.94, p = 0.02)
Wang et al. (2013) [34]	155	Non-small cell lung carcinoma	Beta-blockers improved overall survival (HR 0.78, 95 % CI 0.63–0.97, p = 0.02)
Grytli et al. (2014) [35]	1115	Prostate carcinoma	The use of beta-blockers was not associated with reduced all-cause mortality (HR 0.92, 95 % CI 0.83–1.02)
Choi et al. (2014) [38]	6717	Any	Beta-blocker usage was associated with significantly improved overall survival (HR 0.79, 95 % CI 0.67–0.93, p = 0.004)

Thus, experimental and clinical evidences have indicated the efficacy of beta-blockers for the treatment of cancer. Although there are no clinical trials to clarify it prospectively, to confirm these promising effects of beta-blockers on cancer, two RCTs are ongoing to investigate the preventive role of beta-blockers in patients with breast cancer (NCT 00502684) and CRC (NCT 00888797) undergoing surgery with curative intent.

### ACE inhibitors, ARBs and cancer

**Fig. 3:**
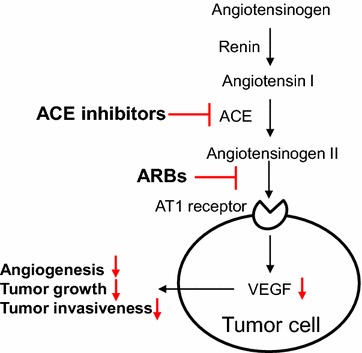
Local RAS, ACE inhibitors and ARBS in tumor cells. In tumor cells, angiotensin II promotes VEGF production via AT1 receptor, resulting in increased angiogenesis. ACE inhibitors and ARBs attenuate local RAS and reduce VEGF-dependent angiogenic signals in cancer

Angiotensin II promotes vasoconstriction and sodium reabsorption via angiotensin II type 1 (AT1) receptors in the renin-angiotensin system (RAS), resulting in increased blood pressure. Both of ACE inhibitors and ARBs, agents blocking RAS, are widely used in the cardiovascular fields for the treatment of heart failure, hypertension and old myocardial infarction. In addition to the systemic RAS, recently much attention has been paid to the existence of the local RAS in tumor cells. Indeed, AT1 receptors have been identified in various types of human cancer, such as renal cell carcinoma [39], laryngeal carcinoma [40], pancreatic cancer [41], ovarian cancer [42], breast cancer [43], melanoma [44], and intrahepatic cholangiocarcinoma [45]. Moreover, immunohistochemical analysis showed co-localization of AT1 receptor and vascular endothelial growth factor (VEGF), a major angiogenic protein, in pancreatic cancer cells [46], and expression of AT1 receptor was detected more frequently in high-grade invasive ovarian cancer than in benign ovarian tumor, and was positively correlated with expression of VEGF [42]. Administration of angiotensin II upregulated VEGF [42, 43, 46] and increased tumor angiogenesis [42], tumor growth [45], and tumor invasiveness [42]. Thus, these findings suggested that the local RAS controlled VEGF and tumor progression, and then blockade of the local RAS has been noted as a promising strategy for the treatment of cancer. Indeed, ACE inhibitors suppressed VEGF expression, VEGF-induced angiogenesis and tumor growth [47, 48] and ARBs also showed similar effects in certain cancer cell lines and animal cancer models [41–43, 45, 46]. Proposed action mechanisms are presented in Fig. 3.

In 1998, an observational study first demonstrated that hypertensive patients taking ACE inhibitors had a reduced cancer risk compared with patients in the control group (RR 0.72, 95 % CI 0.55–0.92) [49]. However, following clinical studies failed to show the favorable effects of ACE inhibitors on cancer risk or outcome [50–54]. For example, Lindholm et al. also investigated a protective role of ACE inhibitors for cancer in elderly patients with hypertension. In this study, ACE inhibitor usage was not associated with decreased risk of new cancer occurrence (standardized incidence ratio 0.99, 95 % CI 0.86–1.13) [52]. Furthermore, in 2010 a meta-analysis of five RCTs revealed that the use of ARBs increased cancer risk (RR 1.08, 95 % CI 1.01–1.16) [55]. However, subsequent several meta-analyses showed no significant association between the use of ARBs and new cancer risk [56, 57] (Table 3). Judging from the results of clinical studies, there is no reason to administer ACE inhibitors or ARBs for the prevention and/or treatment of cancer despite the favorable findings in experimental research. Further long-term prospective trials are needed.

**Table 3:** Anti-tumor effects of ACE inhibitors or ARBs in recent clinical studies

Authors (year), reference	Number of patients taking ACE inhibitors or ARBs	Medication	Type of cancer	Main findings
Ronquist et al. (2004) [54]	100	ACE inhibitors	Prostate cancer	Current use of ACE inhibitors was not associated with decreased risk of prostate cancer (OR 0.9, 95 % CI 0.7–1.1)
Sjoberg et al. (2007) [53]	62 and 101	ACE inhibitors	Esophageal and gastric cancer	Current use of ACE inhibitors did not decrease the risk of esophageal and gastric cancer (OR 0.79, 95 % CI 0.60–1.05 for esophageal cancer; OR 1.11, 95 % CI 0.88–1.39 for gastric cancer)
Sipahi et al. (2010) [55]	2510	ARB	Any	Patients taking ARBs had a significantly increased risk of new cancer development (RR 1.08, 95 % CI 1.01–1.15; p = 0.016)
Pasternak et al. (2011) [56]	3954	ARB	Any	ARB did not increase the risk of cancer (RR 0.99, 95 % CI 0.95–1.03)
The ARB Trialists Collaboration (2011) [57]	4549	ARB	Any	There was no association between ARB usage and cancer incidence (OR 1.00, 95 % CI 0.95–1.04)

### Cardiac glycosides and cancer

**Fig. 4:**
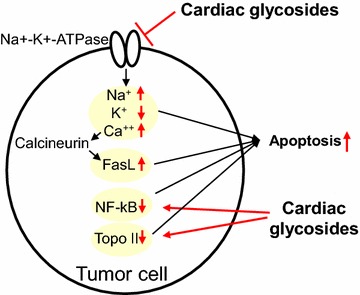
Cardiac glycoside-induced apoptosis in tumor cells. Cardiac glycosides bind to Na^+^-K^+^-ATPase and decrease the membrane potential and increase intracellular Na^+^ and Ca^++^ in certain human cancer cell lines, resulting in activation of calcineurin and transcriptional upregulation of Fas ligand. Cardiac glycosides also suppressthe expression of nuclear factor-kappaB and inhibit DNA topoisomerase II. All of these activities induce apoptosis in human cancer cells

Cardiac glycosides were originally derived from the foxglove. Two types of cardiac glycosides, digoxin and digitoxin, have been currently prescribed to treat heart failure or to reduce heart rate in the cardiovascular field, whereas the relationship between cardiac glycosides and cancer have been noted since Shiratori et al. [58] reported the antiproliferative effect of cardiac glycosides in cancer cells in1967. Sodium- and potassium-activated adenosine triphosphatase (Na^+^-K^+^-ATPase), the primary target of cardiac glycosides, exports three sodium ions in exchange for two potassium ions using ATP as an energy source, thereby maintaining the cell membrane potential. Indeed, increased expression of Na^+^-K^+^-ATPase was found in gastric [59] and bladder cancer cells [60], and elevated Na^+^-K^+^-ATPase activity was observed in highly invasive human renal carcinoma cells [61]. Cardiac glycosides bind to Na^+^-K^+^-ATPase and disrupt its abilities in tumor cells. Namely, cardiac glycosides decreased the membrane potential and increase intracellular Na^+^, which caused the induction of apoptosis in human cancer cells [62], and also increased intracellular Ca^2+^ in human prostate adenocarcinoma cells, resulting in activation of calcineurin and transcriptional upregulation of Fas ligand, which can induce apoptosis [63]. Some other mechanisms of cardiac glycosides such as suppression nuclear factor-kappaB [64] and inhibition of DNA topoisomerase II [65] were also shown to induce apoptosis in tumor cells (Fig. 4). The complex mechanism in cardiac glycoside-induced apoptosis has been already well documented [66–68].

In clinical settings, there were several studies evaluating the effects of cardiac glycosides on the development and progression of cancer. Early observational studies showed that the use of cardiac glycosides reduced the rate of recurrence and the malignant grade of breast cancer [69, 70] and a subsequent 20-year follow-up revealed that patients with cardiac glycosides had a significantly lower mortality rate (6 %) than those without cardiac glycosides (34 %) [71]. These findings seemed to indicate the anti-tumor effect of cardiac glycosides, but the results from recent reports have been inconsistent with them. In 2001, a large cohort study (n = 9271) revealed that patients taking cardiac glycosides had a higher incidence of cancer compared with controls [72]. Moreover, the use of cardiac glycosides was associated with an increased risk of invasive breast cancer (RR 1.30, 95 % CI 1.14–1.48) among post-menopausal women [73] and the incidence of estrogen receptor-positive breast cancer was significantly higher than that of estrogen receptor-negative breast cancer in women taking cardiac glycosides [74]. Cardiac glycosides also increased the risk of corpus uteri cancer (RR 1.48, 95 % CI 1.32–1.65), whereas did not affect the incidence of ovary cancer (RR 1.06, 95 % CI 0.92–1.22) and cervix cancer (RR 1.00, 95 % CI 0.79–1.25) [75] and reduced the risk of prostate cancer (RR 0.76, 95 % CI 0.61–0.95) [76] (Table 4). These findings suggest that the estrogenic effect of cardiac glycosides might play an important role in inhibiting cancer, but further experimental studies and RCTs setting cancer-related events as primary endpoints should be performed to confirm this hypothesis.

**Table 4:** Relationship between cardiac glycosides and the incidence of cancer in recent clinical studies

Authors (year), reference	Number of patients taking cardiac glycosides	Medication	Type of cancer	Main findings
Haux et al. (2001) [72]	9271	Digitoxin	Any	Digitoxin use increased the risk of cancer (SIR 1.27, 95 % CI 1.18–1.37). Plasma digitoxin levels were negatively correlated with the risk of cancer
Ahern et al. (2008) [73]	2890	Digoxin	Breast cancer	Digoxin use was associated with the increased risk of breast cancer (OR 1.30, 95 % CI 1.14–1.48). The risk was positively correlated with the duration of digoxin exposure (OR for 7–18 years of digoxin use 1.39, 95 % CI 1.10–1.74)
Biggar et al. (2011) [74]	104,648	Digoxin	Breast cancer	Current digoxin use increased the risk of breast cancer (RR, 1.39; 95 % CI, 1.32–1.46). In digoxin users, the risk was higher for ER-positive breast cancers (RR, 1.35; 95 % CI, 1.26–1.45) than for ER-negative breast cancers (RR, 1.20; 95 % CI, 1.03–1.40)
Biggar et al. (2012) [75]	104,648	Digoxin	Corpus uteri cancer	Current digoxin use increased the risk of corpus uteri cancer (RR 1.48, 95 % CI 1.32–1.65). (RR 1.06, 95 % CI 0.92–1.22) (RR 1.00, 95 % CI 0.79–1.25)
Biggar et al. (2012) [75]	104,648	Digoxin	Ovary cancer	Current digoxin use increased the risk of ovary cancer (RR 1.06, 95 % CI 0.92–1.22)
Biggar et al. (2012) [75]	104,648	Digoxin	Cervix cancer	Current digoxin use increased the risk of cervix cancer (RR 1.00, 95 % CI 0.79–1.25)
Platz et al. (2011) [76]	936	Digoxin	Prostate cancer	Current digoxin use decreased the risk of prostate cancer (RR 0.76, 95 % CI 0.61–0.95)

### Statins and cancer

**Fig. 5:**
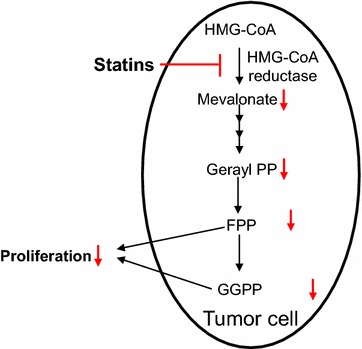
Effects of statins on mevalonate pathway in tumor cells. Downstream products in the mevalonate pathway such as geranylgeranyl pyrophosphate (GGPP) and farnesyl pyrophosphate (FPP) are substrates in protein prenylation. By inhibiting the formation of GGPP and FPP, statins exert anti-proliferatic activity in tumor cells

Statins, competitive inhibitors of 3-hydroxy-3-methylglutaryl-coenzyme A reductase, block the formation of mevalonate in the mevalonate pathway to synthesize cholesterol in liver and are commonly administered for the treatment of hyperlipidemia and the secondary prevention of atherosclerotic diseases such as myocardial infarction and ischemic stroke. Geranylgeranyl pyrophosphate (GGPP) and farnesyl pyrophosphate (FPP) are downstream products of the mevalonate pathway and used as substrates in protein prenylation, which is essential for localization of proteins in cell membranes [77]. As GGPP and FPP are also inhibited by statins, it has been proposed that statins could cause apoptosis in tumor cells (Fig. 5). Indeed, experimental studies have shown that statins suppressed proliferation in multiple human cancer cell lines through this inhibitory activity [78–80]. Despite this promising anti-tumor effect of statins, the results of clinical studies are controversial. Initial clinical trials showed that statins were associated with a significant reduction in overall cancer incidence [81, 82]. Some subsequent meta-analyses have demonstrated that statins significantly reduced the risk of prostate [83], esophageal [84] and gastric cancer [85], while other recent meta-analyses failed to show benefits of statins in cancer-associated mortality [86], and in the risk of lung [87] and skin cancer [88]. The effects of statins on cancer in recently published meta-analyses are presented in Table 5. On the other hand, statins enhanced the efficacy of treatment in acute myeloid leukemia [89] and hepatocellular carcinoma [90], when combined with chemotherapeutic agents. Further clinical investigation should be performed, since in most of clinical studies to investigate the role of statins, the primary endpoint was not set on cancer-related events and the observation period was not long enough to observe the development and/or prevention of cancer.

**Table 5:** Effect of statins on cancer in recent meta-analyses

Authors (year), reference	Number of studies	Type of cancer	Main findings
Bansal et al. (2012) [83]	15 cohort and 12 case–control studies	Prostate cancer	Statins decreased the risk of prostate cancer (RR 0.93, 95 % CI 0.30–0.86) and advanced prostate cancer (RR 0.80, 95 % CI 0.70–0.90)
Singh et al. (2013) [84]	13 studies (including a post hoc analysis of 22 RCTs)	Esophageal cancer	Statins reduced the risk of esophageal cancer (OR 0.72 95 % CI 0.60–0.86)
Wu et al. (2013) [85]	3 post hoc analyses of 26 RCTs and 8 observational studies	Gastric cancer	Statin use was associated with a decreased risk of gastric cancer (RR 0.73, 95 % CI 0.58–0.93)
Emberson et al. (2012) [86]	27 RCTs	Any	Statins did not reduce the incidence of, or mortality from, any type of cancer (RR 1.00, 95 % CI 0.96–1.05 for incidence; RR 1.00, 95 % CI 0.93–1.08)
Tan et al. (2013) [87]	5 RCTs, 7 cohort and 7 case–control studies	Lung cancer	Statin did not decrease the risk of lung cancer either among RCTs (RR 0.91, 95 % CI 0.76–1.09), cohort studies (RR 0.94, 95 % CI 0.82–1.07) and case–control studies (RR 0.82, 95 % CI 0.57–1.16)
Zhang et al. (2014) [88]	29 studies (including a post hoc analysis of 8 RCTs)	Skin cancer	Statins did not reduce the risk of skin cancer among melanoma (RR 0.94, 95 % CI 0.85–1.04) or non-melanoma skin cancer (RR 1.03, 95 % CI 0.90–1.19)

## Repurposing of cardiovascular drugs in pathological conditions other tan cancer

### Propranolol and infantile hemangioma

The efficacy of oral propranolol for infantile hemangioma was first reported in 2008 [91]. Several clinical studies [92–94], including two small RCTs [95, 96], also revealed that infantile hemangioma regressed with the treatment of propranolol. Furthermore, in 2015, a large-scale RCT showed that propranolol at a dose of 3 mg per kg for 6 months was well-tolerated and effective in the treatment of infantile hemangioma [97]. At present, propranolol is regarded as a first-line therapy for infantile hemangioma, though there is room for further research into duration or regimen of propranolol.

### Beta-blockers and migraine

The efficacy of beta-blockers for the prevention of migraine has been evaluated in RCTs. A controlled double-blind trial showed that propranolol was more efficacious than placebo and as efficacious as cyproheptadine in reducing frequency, duration and severity of migraine attacks [98]. Moreover, the efficacy of combination of propranolol and cyproheptadine was greater than that of propranolol or cyproheptadine alone. Subsequently, another controlled double blind trial revealed the more potent effect of metoprolol for the prevention of migraine attacks than aspirin [99]. In this study, treatment effectiveness was defined as a 50 % decrease in the rate of migraine attacks and the response rate was 45.2 % in metoprolol group and 29.6 % in aspirin group respectively. In the current guidelines, propranolol and metoprolol are listed up as effective medications for the treatment of migraine [100].

### Beta-blockers and cirrhosis

In patients with cirrhosis, portal hypertension gradually develops, leading to variceal bleeding, hepatic encephalopathy, ascites, spontaneous bacterial translocation, hepatorenal syndrome. They have hyperdynamic circulatory abnormalities, such as an increased cardiac output and a decreased peripheral vascular resistance. As a result, sympathetic nervous system is activated in this condition. To counteract these abnormalities and reduce portal pressure, NSBB is regarded as effective [101]. Selective beta-1 antagonists were less beneficial for the treatment of cirrhosis [102, 103], which indicates the importance of both beta-1 and beta-2 adrenergic pathways. Recently, carvedilol has been noted as a promising medication for portal hypertension due to its anti-alpha-1 adrenergic activity, which decreases the hepatic vascular resistance, in addition to the beta-blocking activities [104].

In 1981, Lebre et al. first reported the effects of NSBB on the secondary prevention of variceal bleeding in patients with cirrhosis. Since then, many clinical studies have been performed to examine the role of NSBB in patients with chronic liver disease, cirrhosis and portal hypertension. A meta-analysis of 12 RCTs (n = 769) demonstrated that the beta-blocker use reduced the rate of recurrent bleeding and mortality compared to placebo [105]. There is no significant difference between beta-blocker alone and the combination of beta-blocker and nitrate, despite the previous findings indicating the effectiveness of nitrate for the treatment of cirrhosis [106]. In a 2012 meta-analysis, combining NSBB and endoscopic variceal ligation resulted in less recurrent bleeding than either treatment alone [107].

In addition, the use of NSBBs has been considered to be effective for the primary prophylaxis as well as the secondary prophylaxis. A meta-analysis demonstrated that NSBB decreased variceal bleeding and mortality in patients with cirrhosis without previous gastrointestinal bleeding [108]. The current guidelines for the management of portal hypertension, Baveno VI, also recommend that patients with high-risk small varices or large/medium varices should receive NSBB, if not contradictory, or endoscopic variceal ligation for the primary prevention of variceal bleeding [109].

On the other hand, there are few studies to investigate the role of NSBB to prevent the formation of varices. Groszmann et al. [110] demonstrated that NSBB did not prevent the new development of varices in patients with cirrhosis and portal hypertension without varices. The use of NSBB for the pre-primary prophylaxis is not recommended in the current guidelines [109].

In addition, it would be noted that an observational study showed the harmful effect of NSBB in patients with advanced cirrhosis and refractory ascites. In this study, the use of NSBB was associated with significantly poor 1-year survival compared with control (19 vs 64 %, p < 0.0001) [111]. However, further studies should be performed to evaluate the efficacy and safety of NSBB in this population.

### Beta-blockers and osteoporosis

Previous experimental studies revealed that beta-adrenoreceptors are expressed on osteoblastic and osteoclastic cells and that a beta agonist stimulates osteoblasts resulting in bone resorption [112]. These findings supported that a beta agonist decreased bone mass, while a beta antagonist increased bone mass in mice [113].

Recently the importance of sympathetic nervous system in bone remodeling has been focused on. Ducy et al. [114] demonstrated that intracerebroventricular infusion of leptin inhibited bone formation and decreased bone mass in mice, which indicated the central role of leptin in bone remodeling. Furthermore, Takeda et al. [113] showed that the effect of leptin was mediated by beta2-adrenoreceptors on osteoblasts. Namely, blockade of beta2-adrenoreceptors caused bone formation [113], while stimulation of beta2-adrenoreceptors resulted in bone resorption [115]. These findings suggest that beta-blockers could be a therapeutic option for osteoporosis.

There are numerous clinical studies which evaluated the effects of beta-blockers on the risk of osteoporotic fractures in humans. The results are inconsistent, however recent meta-analyses have shown that beta-blockers reduced the risk of fracture by approximately 15 %, independent of gender, fracture site and dose [116, 117]. The most recent meta-analysis (n = 1,644,570) has demonstrated that beta-blocker use was associated with a significantly lower risk of fractures (16 studies, RE pooled ES = 0.86, 95 % CI 0.78–0.93) [118]. Intriguingly, beta1-selective beta-blockers significantly reduced the risk of any fracture compared with NSBB (6 studies, RE pooled ES = 0.82, 95 % CI 0.69–0.97) [118]. These findings do not support the proposed mechanism in which bone formation is promoted by the blockade of beta2-adrenoreceptor.

Additionally, hemodynamic alteration with beta-blockers might affect the incidence of falls and fractures. Namely, anti-arrhythmic actions could prevent subjects, especially elderly people, from a fall, while (orthostatic) hypotension and/or bradycardia could make them injured in a fall, sometimes a hip-fracture. Although findings of clinical studies have been inconsistent with regard to beta-blockers and risk of falls, there are no evidence that beta-blockers increased the incidence of falls [119–121].

Collectively, the roles of beta-adrenoreceptors in bone remodeling are complex and further studies are needed to clarify them. Asbeta-blockers appear to reduce the risk of osteoporotic fracture in clinical settings, RCTs should be performed to evaluate the efficacy and safety of beta-blockers in this condition.

### Losartan and Marfan’s syndrome

Marfan’s syndrome is caused by mutations in the gene encoding fibrillin-1 [122], which regulates the transforming growth factor beta (TGF-beta) signaling pathway. In a mouse model of Marfan’s syndrome, fibrillin-1 deficiency was associated with increased TGF-beta signaling [123, 124], which is thought to contributes to the development of aortic aneurysm. In 2006, Habashi et al. [125] showed that losartan, one of the ARBs, suppressed the excessive TGF-beta signaling and prevented the formation of aortic aneurysm in fibrillin-1 deficient mice. In this study, the losartan treatment was significantly efficacious for the prevention of aortic aneurysm compared with the propranolol treatment [125]. Subsequently, the effect of losartan was examined in patients with Marfan’s syndrome. Two small cohort studies demonstrated that losartan significantly slowed the rate of aortic growth in pediatric patients with Marfan’s syndrome [126, 127] and in a RCT, aortic dilation rate was reduced by losartan in adult patients with Marfan’s syndrome [128]. However, a recent RCT showed that there was no significant difference in the progression of aortic diameter between losartan-treated and atenolol-treated patients with Marfan’s syndrome [129]. Further experimental and clinical investigation should be performed, though the suppression of enhanced TGF-beta signaling seems important in this condition.

### Preoperative statins and perioperative risk

Experimental research has demonstrated that statins improve endothelial function, attenuate vascular and myocardial remodeling, inhibit vascular inflammation and oxidation, and stabilize atherosclerotic plaques as pleiotropic effects beyond cholesterol lowering [130], which are expected to prevent plaque rupture and subsequent cardiovascular events in the perioperative period. Indeed, several observational studies have suggested that statin use decreased short-term mortality and myocardial infarction [131–134]. In addition, a RCT showed that atorvastatin was associated a significant reduction in the incidence of cardiac events at 6 months follow-up after vascular surgery [135]. In the light of these findings, both the current ESC/ESA and ACC/AHA guidelines give a class I recommendation for continuing perioperative statins [136, 137]. In statin-naïve patients, two meta-analyses showed the preventive effect of statins on perioperative myocardial infarction and death, which allows a class IIa recommendation in the ESC/ESA and ACC/AHA guidelines for pre-operative initiation of statins for patients undergoing vascular surgery [136, 137]. In non-cardiac surgery, evidence is insufficient, but statins appear more beneficial in patients with accumulated cardiovascular risk and statins are often prescribed in such case in clinical practice.

### Statin and contrast-induced nephropathy

Contrast-induced nephropathy (CIN) is characterized by acute kidney injury, caused by contrast medium. To prevent CIN, there are limited strategies such as volume expansion, use of iso-osmolar contrast and less amount of contrast media. The pathophysiological mechanisms of CIN are not fully understood, but contrast media might cause renal vasoconstriction, oxidative stress, inflammation and direct tubular necrosis [138]. As statins improve endothelial function and exert anti-inflammatory and antioxidative properties as well as lower cholesterol [139–141], statins have been regarded as promising therapeutic approach. Experimental research suggested that statins ameliorated acute ischemic renal failure and prevented tubular necrosis in rats [142]. In clinical setting, a recent meta-analysis seven RCTs showed that the use of high-dose statins significantly reduced the risk of CIN compared to that of low-dose statins or placebo (RR 0.51, 95 % CI 0.34–0.76) [143]. In addition, a large-scale RCT, Novel Approaches for Preventing or Limiting Events (NAPLES) II trial, showed that a single high-dose of atorvastatin significantly reduced the incidence of CIN compared with placebo in patients with chronic kidney disease undergoing elective coronary angiography (OR 0.22, 95 % CI 0.07–0.22) [144]. Another large-scale RCT, protective effect of rosuvastatin and antiplatelet therapy on contrast-induced acute kidney injury and myocardial damage in patients with acute coronary syndrome (PRATO-ACS) trial, randomly assigned patients with acute coronary syndrome undergoing an early coronary angiography to rosuvastatin or placebo, and revealed that high-dose rosuvastatin prevented CIN (OR 0.38, 95 % CI 0.20–0.71) [145]. These findings support the use of statins for the prevention of CIN in coronary angiography, while future research should investigate effect of statins in other examinations or treatments such as computed tomography, as well as timing of initiation and duration of statin treatment.

### Minoxidil and androgenic alopecia

Oral minoxidil had been originally developed as an antihypertensive drug in 1960s and was reported to cause hypertrichosis as well as an antihypertensive effect in 1970s/80s [146, 147], which led to the research of topical minoxidil for the treatment of alopecia [148]. Several mechanisms how minoxidil improves alopecia have been proposed. Minoxidil stimulates cutaneous blood flow through local vasodilatory effect in human scalps [149], up-regulates the expression of VEGF in human hair dermal papilla cells [150], and prolongs the anagen period of hair follicle through the opening of potassium channels [151]. All of these actions could help to promote or maintain hair growth.

In male alopecia, 2 and 5 % minoxidil solutions have been approved and the 5 % solution has shown higher efficacy than the 2 % solution [152]. Recently, the foam type has been newly developed, and the effect of 5 % minoxidil foam has been comparable to the 5 % minoxidil solution. The minoxidil treatment during 5 years has shown the continuous efficacy [153], while the therapeutic effect has disappeared in 24 weeks after discontinuation [154]. In female alopecia, only the 2 % minoxidil solution or foam are currently used. As higher dose seems to show higher efficacy both in males and females, further clinical studies might allow higher dose to be approved.

## Conclusions

A number of studies have demonstrated anti-tumor activities of cardiovascular drugs in tumor cells and animal models, while findings of clinical trials, including large-scale RCTs, have often been inconsistent with those of preclinical studies or other clinical trials. Meta-analysis might some contributions to this ‘dissociation’. Meta-analysis is convenient and widely used, while we should pay appropriate attention to the fact that meta-analysis is hard to gather detailed data such as patient characteristics and easily biased. It could be important to consider cancer type, cancer stage and patient characteristics such as age, sex, body mass index in evaluating cancer. Regarding aspirin and beta-blockers, RCTs are ongoing and the results are anticipated with great interest.

In the pathophysiological conditions other than cancer, some cardiovascular drugs have already obtained new indications, such as propranolol for infantile hemangioma, beta-blockers for migraine, and minoxidil for androgenic alopecia. Preoperative use of statins for perioperative risk reduction may be used for patients undergoing non-cardiovascular surgery.

Thus, based on the available knowledge and information, it would be expected that unknown mechanisms of action of drugs are investigated by experimental studies and that clinical evidences are established by well-organized RCTs. Close link between experimental and clinical studies is essential.

## Abbreviations

ACE: angiotensin converting enzyme
ARBs: angiotensin II receptor blockers
AT1: angiotensin II type 1
cAMP: cyclic adenosine monophosphate
CI: confidence interval
CIN: contrast-induced nephropathy
COX: cyclooxygenase
CRC: colorectal cancer
FAK: focal adhesion kinase
FPP: farnesyl pyrophosphate
GGPP: geranylgeranyl pyrophosphate
HR: hazard ratio
Na^+^-K^+^-ATPase: sodium- and potassium-activated adenosine triphosphatase
NSBB: non-selective beta-blockers
OR: odds ratio
PGE2: prostaglandin E2
PKA: protein kinase A
RAS: renin-angiotensin system
RCTs: randomized controlled trials
RR: risk ratio
TGF-beta: transforming growth factor beta
TXA2: thromboxane A2
VEGF: vascular endothelial growth factor
